# The Feasibility of Studying Metabolites in PICU Multi-Organ Dysfunction Syndrome Patients over an 8-Day Course Using an Untargeted Approach

**DOI:** 10.3390/children8020151

**Published:** 2021-02-18

**Authors:** Mara Leimanis-Laurens, Danny Gil, Andrew Kampfschulte, Claire Krohn, Elizabeth Prentice, Dominic Sanfilippo, Jeremy W. Prokop, Todd A. Lydic, Surender Rajasekaran

**Affiliations:** 1 Pediatric Critical Care Unit, Helen DeVos Children’s Hospital, 100 Michigan Street NE, Grand Rapids, MI 49503, USA; dangil2480@yahoo.com (D.G.); elizabeth.prentice@helendevoschildrens.org (E.P.); dominic.sanfilippo@helendevoschildrens.org (D.S.); surender.rajasekaran@spectrumhealth.org (S.R.); 2 Department of Pediatrics and Human Development, College of Human Medicine, Michigan State University, Life Sciences Building, 1355 Bogue Street, East Lansing, MI 48824, USA; prokopje@msu.edu; 3 Spectrum Health, 100 Michigan Street NE, Grand Rapids, MI 49503, USA; andrew.kampfschulte@spectrumhealth.org; 4 College of Human Medicine, Michigan State University, 15 Michigan Street NE, Grand Rapids, MI 49503, USA; krohncla@msu.edu; 5 Department of Pharmacology and Toxicology, Michigan State University, 1355 Bogue Street, East Lansing, MI 48824, USA; 6 Department of Physiology, Collaborative Mass Spectrometry Core, Michigan State University, 578 S. Shaw Ln. Chemistry Building, East Lansing, MI 48824, USA; lydictod@msu.edu

**Keywords:** blood plasma, extracorporeal membrane oxygenation, metabolites, multiple organ dysfunction syndrome, pediatric intensive care unit, liquid chromatography–mass spectrometry

## Abstract

Metabolites are generated from critical biological functions and metabolism. This pediatric study reviewed plasma metabolites in patients suffering from multi-organ dysfunction syndrome (MODS) in the pediatric intensive care unit (PICU) using an untargeted metabolomics approach. Patients meeting the criteria for MODS were screened for eligibility and consented (*n* = 24), and blood samples were collected at baseline, 72 h, and 8 days; control patients (*n* = 4) presented for routine sedation in an outpatient setting. A subset of MODS patients (*n* = 8) required additional support with veno-atrial extracorporeal membrane oxygenation (VA-ECMO) therapy. Metabolites from thawed blood plasma were determined from ion pairing reversed-phase liquid chromatography–mass spectrometry (LC-MS) analysis. Chromatographic peak alignment, identification, relative quantitation, and statistical and bioinformatics evaluation were performed using MAVEN and MetaboAnalyst 4.0. Metabolite analysis revealed 115 peaks per sample. From the partial least squares-discriminant analysis (PLS-DA) with variance of importance (VIP) scores above ≥2.0, 7 dynamic metabolites emerged over the three time points: tauro-chenodeoxycholic acid (TCDCA), hexose, *p*-hydroxybenzoate, hydroxyphenylacetic acid (HPLA), 2_3-dihydroxybenzoic acid, 2-keto-isovalerate, and deoxyribose phosphate. After Bonferroni adjustment for repeated measures, hexose and *p*-hydroxybenzoate were significant at one time point or more. Kendall’s tau-b test was used for internal validation of creatinine. Metabolites may be benign or significant in describing a patient’s pathophysiology and require operator interpretation.

## 1. Introduction

The ability to identify, quantify, and analyze the metabolic profile of a pediatric patient allows us to investigate the interaction between both physiological and pathologic states. Metabolites are under the control of environmental pressures, such as nutrition [1], viral infections (such as Covid-19 [2], Ebola [3]), gut bacterial composition and cancer [4], medications [5,6], and a patient’s own pre-existing genetic make-up [7]. Metabolites, being low molecular weight molecules and/or products of metabolic pathways, have been growing in appeal medically over the last decade for their potential in disease characterization, drug discovery, and precision medicine [8,9].

We have previously described the current cohort of patients for patient whole blood transcriptomics [10,11] and plasma lipidome [12]. This has revealed a complex biology in a heterogenous patient population with a non-uniform patient response to treatments over an 8-day course (stabilization and recovery phases) of illness during pediatric intensive care unit (PICU) admission. Complementary to these previously reported analytic modalities from whole blood [10,11,12], the aim of this current report was threefold: (1) to determine the feasibility of undertaking blood plasma metabolite work in the PICU setting, (2) characterize total blood plasma metabolites (polar, charged) using an untargeted approach, and (3) to determine change in metabolites over an 8-day PICU course. There is a gap in our understanding of the complex interaction between pediatric critical illness, specifically multi-organ dysfunction syndrome (MODS) [13] (affecting 20% of PICU admissions [14], resulting in 10 times the mortality rate [15]), and their respective blood metabolites.

## 2. Materials and Methods

### 2.1. Study Population, Site, and Sample Collection

After Institutional Review Board approval, a short-term longitudinal design was adopted at Helen DeVos Children’s Hospital (2016-062-SH/HDVCH). Samples were collected under the protocol and study design [10,11,12] in a quaternary-care urban pediatric hospital in Western Michigan. In brief, patients who were identified as having MODS were enrolled, 24 in total, with an additional 4 sedation-control patients. These 24 patients were then further classified as needing veno-arterial extracorporeal membrane oxygenation (VA-ECMO) as a therapeutic modality (*n* = 8) according to Extracorporeal Life Support Organization (ELSO) criteria [16]. Blood samples from the patients were obtained and placed into EDTA-filled tubes, and plasma was processed and stored at −80 °C for later use. All samples had undergone one freeze–thaw before processing and analysis.

### 2.2. Metabolite Extraction and Liquid Chromatography-Mass Spectrometry (LC-MS)

Plasma samples (~50 μL) were subjected to biphasic extraction using chloroform/methanol/water as described previously [17] to remove non-polar matrix interference and recover polar metabolites in the aqueous extraction phase. Stable isotope labeled (D^4^)-succinate was added to plasma during extraction for use in estimation of metabolite recovery and for relative quantitation across experimental groups. Samples were filtered through 0.2-μm syringe filters (Fisher Scientific, Hampton, NH, USA) and reconstituted in 100 μL of 50% methanol for use in ion pairing reversed-phase LC-MS analysis.

Targeted polar metabolite identification utilized a Thermo Scientific model TSQ Vantage triple quadrupole mass spectrometer operating in negative ion mode. The mass spectrometer was coupled to Shimadzu Prominence high-performance liquid chromatography equipment (HPLC) with a thermostatic column oven and autosampler. Ten μL sample injections were subjected to gradient elution with (A) 10 mM tributylamine and 15 mM acetic acid (pH 4.95), and (B) methanol according to Luo et al. [18], with separation of metabolites achieved on a Phenomenex Synergi Hydro-RP C18 column (2.0 mm × 150 mm, 3 μm particles, 80 Å pore size). The column was protected by a Phenomenex guard cartridge of identical chemistry. Metabolites were identified and quantitated by selected ion monitoring. Detection parameters for each precursor/product ion pair of interest were optimized using commercially available standards.

### 2.3. Data Analysis

LC-MS data analysis of chromatographic peak alignment, compound identification, relative quantitation, and statistical evaluation across experimental groups was performed using MAVEN software [19]. Only relative quantitation of analytes against a selected internal standard was performed for a comparison of values across experimental treatment groups. “Absolute” quantitation was not carried out in these experiments. Metabolites, as listed in Supplemental Table S1, were initially categorized as anabolic vs. catabolic and endogenous vs. exogenous according to the Human Metabolite Database (HMDB) (https://hmdb.ca/metabolites/) (accessed on 3 December 2020) (Supplemental Figure S1).

**Figure 1:**
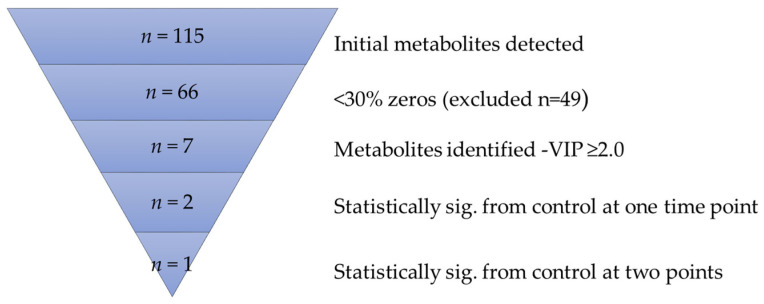
Overview: untargeted analysis. VIP: Variable importance in projection.

Metabolic profiles for sedation-controls were compared with MODS or ECMO patients, quantified as a percentage of the total. Metabolites with >30% of cases with zero values were excluded from further analysis; consequently, 66 metabolites were analyzed over the 3 time points (Figure 1). In MetaboAnalyst 4.0 [20], data were normalized using pareto scaling (mean-centered and divided by the square root of standard deviation of each variable) and subjected to a multivariate partial least squares-discriminant analysis (PLS-DA) [21], using Q2 values for cross-validation [22]. No data points were excluded. To assess the significance of class discrimination, a permutation test was performed and the PLS-DA model was built (between the data (X) and the permuted class labels (Y)) using the optimal number of components as determined by cross-validation for the model based on the original class assignment [23]. This guided analysis allowed for the display of each specific group assignment. Variable importance in projection (VIP) is a weighted sum of squares of the PLS loadings considering the amount of Y-variation explained in each dimension.

Univariate analysis was performed using MedCalc (MedCalc Software Ltd., Ostend, Belgium) for candidate metabolites as determined by VIP scores >2.0 from 7 metabolites, using independent *t*-tests (equal variances) and Welch’s test (unequal variances). A Bonferroni correction of *p*-value (<0.008) was used to identify statistically significant associations with metabolites (to control for Type I errors), which was calculated by dividing the significance threshold of 0.05 by the number of repeated measures; in this case, MODS and ECMO compared with sedation-controls (at baseline, time 72 h, and 8 days). Box and whisker plots were generated for the 2 remaining metabolites of interest, which included the median, the interquartile range (box), the outer range (whiskers) to pictorially summarize the central tendency, dispersion, skewness, and extremes of the dataset [24].

## 3. Results

### 3.1. Metabolite Ontology and Origin

All 115 metabolites are listed in Supplemental Table S1 according to compound identification from the metabolic mass to charge ratio and retention time. From here, we looked at the ontology of the metabolites in order to characterize them according to any known function and origin. We found that the majority of metabolites detected were endogenous in nature (76%) and associated with catabolic (38%), anabolic (35%), both catabolic and anabolic metabolism (14%), or unspecified mechanisms of action (13%) (Supplemental Figure S1A,B), according to HMDB (https://hmdb.ca/metabolites/) (accessed on 3 December 2020).

### 3.2. Bioinformatic Analysis

**Figure 2:**
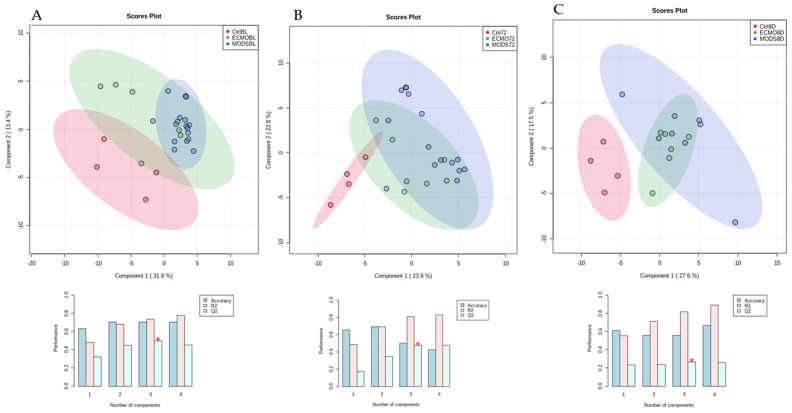
Partial least squares-discriminant analysis (PLS-DA) plot of all patient samples at baseline (**A**), 72 h (**B**), and 8 days (**C**). PLS-DA classification using a different number of components. The red star indicates the best classifier.

An analytical flow chart is presented in Figure 1. The total percentage of metabolites and change over time were visualized using supervised PLS-DA, which revealed clustering of ECMO patients within the MODS patients over the three time points, as compared with the sedation-control group (Figure 2A–C). These groupings were also visualized by heatmap analysis (Supplemental Figure S2A–C), supporting the initial finding of the PLS-DA, whereby the sedation-control patients were found to cluster amongst themselves.

**Figure 3:**
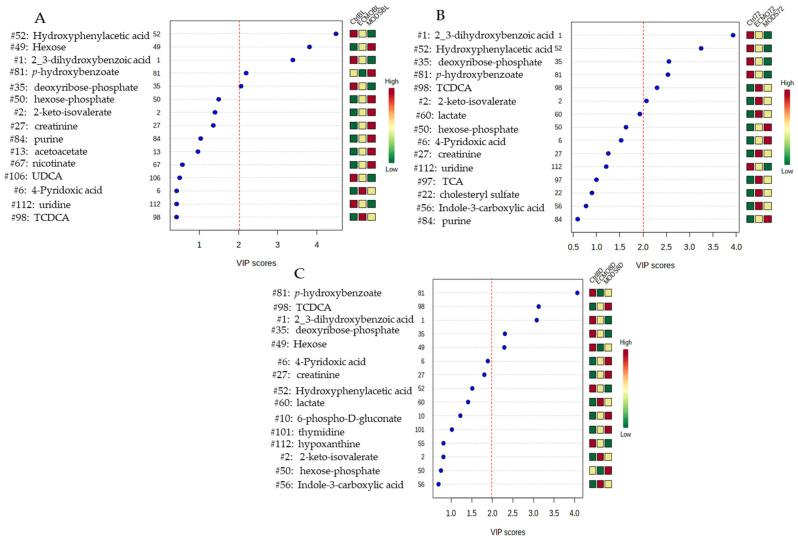
Important features identified by PLS-DA at baseline (**A**), 72 h (**B**), and 8 days (**C**). The colored boxes on the right indicate the relative concentrations of the corresponding metabolite in each group under study. TCDCA: tauro-chenodeoxycholic acid.

The important features as identifies by the PLS-DA at baseline (Figure 3A), at 72 h (Figure 3B), and at 8 days (Figure 3C) reveal seven metabolites with relative concentrations of the corresponding metabolite in each group under study. In total, seven metabolites of interest emerged over the three time points: tauro-chenodeoxycholic acid (TCDCA) (a conjugated bile acid), hexose-monosaccharide (simple sugar), *p*-hydroxybenzoate (biocide-antimicrobial agent/tyrosine, tryptophan, phenylalanine metabolite), hydroxyphenylacetic acid (HPLA) (a metabolite of phenylalanine), 2_3-dihydroxybenzoic acid (drug metabolite), 2-keto-isovalerate (cellular intermediate for the synthesis of branched-chain amino acids), and deoxyribose phosphate (a pentose phosphate).

Starting with HPLA (with a VIP score ≥1.5 at 8 days) over the three time points, the sedation-control values were consistently highest, with ECMO patients demonstrating an intermediate profile and MODS patients having the lowest values according to the relative concentrations. 2_3-Dihydroxybenzoic acid and deoxyribose phosphate share similar relative concentration profiles to HPLA, with the values highest for sedation-control patients compared with MODS with the lowest relative concentrations.

In the exact opposite profile is hexose, which revealed the highest relative concentrations values in MODS patients as compared with the sedation-controls, again with ECMO sharing intermediate profiles at baseline. By Day 8, however, this had changed completely, whereby sedation-controls had the highest relative concentrations, and ECMO the lowest, with MODS demonstrating intermediate profiles. We may extrapolate from this that the patients with critical illness demonstrate some fluctuations in hexose over time, and that the detection of this blood plasma metabolite is a dynamic process. Patients were neither hyper- nor hypoglycemic according to their clinical glucose levels (also a 6-carbon sugar; data not shown), and this is closely monitored at the PICU bedside, given that blood glucose levels have been previously demonstrated to adversely affect patient outcomes, especially in the case of hyperglycemia [25].

The remaining metabolites of interest include *p*-hydroxybenzoate, with the exception of the 72-h time point, revealed that ECMO patients had lower relative concentrations than MODS patients, and TCDCA, which was consistently lower in sedation-control patients as compared with both MODS and ECMO. TCDCA is a marker of liver injury, which was still elevated after 8 days for MODS and ECMO patients. It is believed that liver shock, which is common in this patient population, usually subsides after a few days; from our results, we may speculate that this metabolite was still present at the eighth day after study enrollment. Lastly, keto-isovalerate, like hexose and TCDCA, had higher relative concentrations for both MODS and ECMO patients compared with sedation-controls at both baseline and 72 h (at 8 days, they are similar patterning, however; VIP score <1.0). Additional metabolites of note include lactate (VIP score ≥1.5), which would be expected in this group of patients [24], which was highest in ECMO patients, second in MODS, and lowest in the sedation-controls at the 72-h time point.

### 3.3. Repeated Measures over Three Time Points

**Figure 4:**
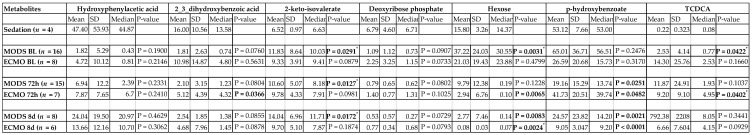
Repeated measures summary statistics for variance of importance (VIP) ≥2.0 at baseline, 72 h and 8 days. Independent *t*-tests were performed (assuming equal variances). * *F*-test for equal variances was *p* ≤ 0.05; Welch’s test (assuming unequal variances) was used; *p*-values less than 0.008 were deemed significant after Bonferroni adjustment, comparing multi-organ dysfunction syndrome (MODS) and extracorporeal membrane oxygenation (ECMO) samples with sedation-controls. TCDCA: tauro-chenodeoxycholic acid.

Furthermore, it was of interest to determine whether any of those metabolites identified by PLS-DA with high VIP scores were statistically significant over time, as this may provide additional understanding and potential biomarker identification of this cohort of untargeted metabolites. When compared with to sedation-controls and corrected for Bonferroni adjustment for repeated measures (*p*-value < 0.008), both hexose and p-hydroxybenzoate were significant at, at least at one time point (Figure 4).

**Figure 5:**
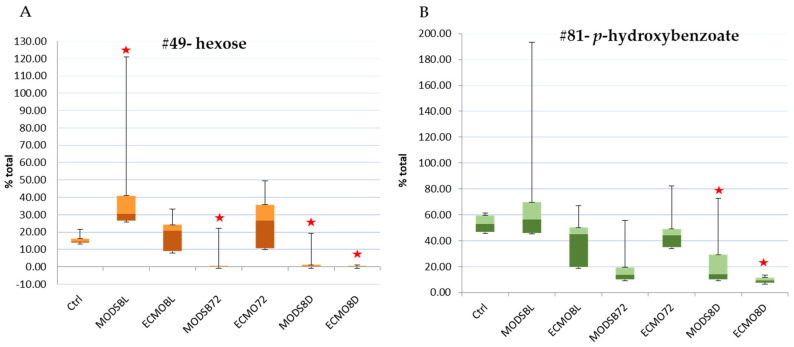
(**A**) hexose; (**B**) p-hydroxybenzoate. The top two metabolites with significant differences from sedation-controls over 8 days. Box and whisker plots, which include the median, the interquartile range (box), and the outer range (whiskers), pictorially summarize the central tendency, dispersion, skewness, and extremes of the dataset using a linear scale. MODS: multi-organ dysfunction syndrome; ECMO: extracorporeal membrane oxygenation; BL: baseline; 72: 72-h time point; 8D: 8-day time point. Stars denote statistical significance from independent *t*-tests or Welch’s test as compared with the sedation-controls.

Box and whisker plots were generated to be able to visualize the distribution of samples over time (Figure 5). From these, we can visualize a large spread of patient blood plasma metabolite values and see that the values change over the three time points. More frequent and intensive sampling would be necessary to determine exact distribution for the acute, stabilization, and recovery phases of MODS and ECMO patients, using a metabolic platform. This illustrates the complexity and the dynamic nature of sampling for this patient population.

### 3.4. Internal Validation

Kendall’s tau-b (τb) test was used for internal validation and revealed a positive correlation between creatinine and the respective creatinine metabolite at all three time points (baseline: τb = 0.708, *p* = 0.000; 72 h: τb = 0.511, *p* = 0.001; 8 days: 0.684, *p* = 0.001) (Supplemental Figure S3). While the three coefficients yielded similar results, the skewness and heteroskedasticity of the data violated the assumptions for Pearson’s *r*. Spearman’s Rho and Kendall’s Tau are both non-parametric and acceptable to use in this case. Spearman’s Rho measures the rank correlation (how the ranks of the *x* and *y* values align), and Kendall’s Tau measures the percentage of concordant pairs, which is also based on ranks but considered to be a more robust measure. Creatinine was found to be high at baseline, which correlated with clinical creatinine values.

## 4. Discussion

We utilized an untargeted LC-MS-based metabolomics approach and therefore did not search for any particular metabolites, but simply sought to identify and test those with a significant correlation to MODS. Multivariate statistical techniques are therefore well suited to an analysis of untargeted metabolomics data. MetaboAnalyst is a web-based platform created and developed by the Wishart group from the University of Alberta, offering R-based software for public use [20,26], and was the software of choice for this analysis.

Much research has been done on biomarkers in patients with sepsis and organ failure in the last two decades, and yet very few, if any, have made the transition to clinical practice [27,28,29]. The goal here was to determine, firstly, if there were any metabolites that stood out in an untargeted approach. Second, if the metabolites could be linked directly to metabolic cycles, then one could ascertain which pathways were being strained then one could develop therapeutic options that could ameliorate those perturbations. This approach may have more utility for the clinician in the future, as he/she can relate this directly to the patient’s clinical condition.

The definition for MODS was taken from the original work of Proulx et al. [13], which is still being used today, in which MODS patients are clinically identified with two or more organs in failure. The detailed pathology of the patients was described in Prokop et al. [10] and the demographic data for this patient cohort was described in Shankar et al. [11], which outlined specifically which organ systems were affected. This being said, all of the patients required vasopressors and mechanical ventilation at the time of patient recruitment, as they were in respiratory failure. The majority of patients had additional organ failure which was renal or liver failure, with a minority with neurological failure. Additional information is listed at 72 h and 8 days in the supplemental material of Shankar et al. [11]; in brief, most patients were in stabilization and recovering by the later time points, based on the routine clinical tests.

From these preliminary results, we may glean a few main findings: the metabolites of MODS and ECMO patients: (1) contain both Phase I and Phase II metabolites; (2) are dynamic in nature; (3) contain both potentially clinically relevant findings (such as TCDCA), as well as those of benign (±inert) function, as normally metabolized and excreted through downstream organ systems (renal, digestive); and (4) can be measured and qualified in the blood plasma of critically ill pediatric patients using an untargeted approach, which correlated to clinical values for routine care (e.g., creatinine).

Phase I and Phase II metabolites were detected amongst the 66 values analyzed. Recent evidence suggests that the gut microbiome influences blood metabolites [30], which undergo Phase I metabolism through oxidation (R-OH), reduction (R-SH), or hydrolysis (R-NH_2_) and Phase II metabolism through sulfation (R-SO_3_H), glucuronidation (R-Gluc), and glutathione conjugation (R-Gl). This may suggest that there are greater metabolic influences beyond the scope of this study that need to be controlled for in future work, such as including gut microbiome profiles.

*p*-Hydroxybenzoate, is thought to be produced via two major pathways: (1) microbial oxidation of petroleum derivative toluene into *p*-hydroxybenzoate, as described for *Pseudomonas* species [31]; and (2) the *de novo* bioproduction of *p*-hydroxybenzoate from amino acids (tyrosine, tryptophan, and phenylalanine) through an intermediate chorismite, via the enzyme chorismite lyase (ubiC) [32]. *p*-Hydroxybenzoate (a paraben and alkyl ester derivative) is commercially used as a preservative and antimicrobial agent in the pharmaceutical and cosmetic industry [33] and therefore may be from an exogenous source. The second possibility is an endogenous source, which has been described in *Escherichia coli* [34] and *Mycobacterium tuberculosis* [35], produced from glucose, which can be toxic at higher concentrations. Lower levels of *p*-hydroxybenzoate, as observed in our MODS and ECMO patients at Day 8, is a Phase I metabolite and may be an indication of gut bacteria dysbiosis (impaired microbiota) and, to date, has not been described in this patient population.

The most dynamic metabolite described herein is hexose, which reflects the fluctuating energetic state of the patients. Low hexose at the third time point (8 days after MODS diagnosis and into their PICU admission) could be a sign of energy deficiency; however, we know that the patients were under close monitoring and, by 72 h, were all receiving some nutritional intervention [12], after largely being nil per os at baseline. Blood glucose control has been further evaluated in Covid-19 patients and is of ongoing concern, given reports of higher mortality and multi-organ injury [36]. HPLA, a phenylcarboxylic acid, has been speculated to be a marker of sepsis in adult cardiac surgery; however, this requires further validation [37].

Cholesterol breaks down in the liver to produce primary bile acids, one being chenodeoxycholic acid, which, together with taurocholic acid, produces the conjugated bile acid TCDCA and is excreted in the intestine, constituting our enterohepatic circulation. In spite of normalizing liver enzymes (alanine transaminase (ALT) aspartate transaminase (AST)) in this patient population, as previously reported [11], TCDCA remained elevated as compared with sedation-controls at the 8-day time point. This indicates that the metabolic profile may illustrate a different landscape on patient recovery, and metabolites may be organ-specific.

The clinical care of patients with MODS is complex and not evidence-based but driven purely by the physician’s “gestalt”. As we have built computational capabilities over the last few years, this clinical utility of these types of dataset needs to be explored. This is considered pilot data, the fact that there were significant findings indicates that there may be reason to move further with future work. This work remains challenging, in part because the patient ages varied significantly. Patients were as young as a few days old, and researchers respected all ethical guidelines which stipulate no more than 2 mL/kg volume (with no more than two blood draws in a 7-day period) for minimal risk studies. This restricted our ability to open the doors on more biochemical pathways that may have required more frequent sampling. We chose to restrict the multiple omics analysis to lipids [12] (which described the patients’ nutritional intake in detail) and metabolites. For future studies, a different strategy could be adopted, whereby looking at metabolites would enable a deeper depth of coverage, with the potential of tailoring the analysis for this population, given the limited sample volumes.

The long-range vision for this work, once we have tested for feasibility, and validated the work in subsequent cohorts, will be to focus on a single or multiple biomarker, using a targeted approach with diagnostic, prognostic, or monitoring ability; future in-house analytic capabilities; and providing real-time clinical support.

We were only able to determine relative quantitation because this was an untargeted approach with much of these metabolites yet to be standardized and absolute quantitation not possible. This being said, in working with infants although a volume of 300 μL is preferred for metabolite work, volumes as low as 50 μL could provide some data outputs, according to our findings. The limitations of the work include a low sample volume, he capture of high-abundance metabolites, and sample integrity that may have been compromised by a previous freeze–thaw cycle. Work is currently ongoing to support a second cohort study, as this patient population has been deemed an understudied vulnerable population of national priority.

## 5. Conclusions

It is feasible to measure blood plasma metabolites in pediatric patients with MODS and undergoing ECMO treatment. Routine laboratory testing in a PICU setting may not reveal ongoing organ-specific impairment or recovery (such as in the case of TCDCA). Current investigations are underway to explore, in more detail, specific organ damage (renal) in the same patient cohort. Metabolites may be benign or significant in describing a patient’s pathophysiology, fluctuate over time, and require operator interpretation.

## Data Availability

Data available upon request.

## Supplementary Materials

The following are available online at https://www.mdpi.com/2227-9067/8/2/151/s1. Table S1: Metabolites identified (*n* = 115); metMz (mass over charge ratio), metRt (retention time), compound name, and compound ID are listed. Figure S1: (a) Metabolic actions of 115 metabolites identified; (b) ontological sources of 115 metabolites identified, taken from Human Metabolite Database (HMDB) (https://hmdb.ca/metabolites/) (accessed on 3 December 2020). Figure S2: Clustering results shown as heatmaps at baseline (A), 72 h (B), and 8 days (C). Figure S3: Correlation of clinical creatinine to untargeted metabolite value(s).

## Abbreviations

HMDB	Human Metabolite Data Base
HPLA	hyroxyphenylacetic acid
HPLC	high-performance liquid chromatography
LC-MS	Liquid chromatography–mass spectrometry
MODS	multi-organ dysfunction syndrome
PICU	pediatric intensive care unit
PLS-DA	partial least squares-discriminant analysis
VA-ECMO	veno-atrial extracorporeal membrane oxygenation
VIP	variance of importance
TCDCA	tauro-chenodeoxycholic acid
