# Supplementary material for: The Feasibility of Studying Metabolites in PICU Multi-Organ Dysfunction Syndrome Patients over an 8-Day Course Using an Untargeted Approach
Source: Children (Basel). 2021 Feb 18;8(2):151. doi: 10.3390/children8020151 (PMC7922853; doi:10.3390/children8020151)
Supplement: Supplementary file 1 [file children-08-00151-s001.pdf]

children

MDPI

Supplementary Materials:

Table 1. Metabolites identified n=115, metMz-*mass over charge*, metRt-*retention time*, compound name, and compound ID listed.

Units are picomole/microliter									
coded	medMz	medRt	compound	compoundId	coded	medMz	medRt	compound	compoundId
1	153.02	1.98	2_3-dihydroxybenzoic acid	C00196	59	207.08	3.52	Kynurenine	C00328|C01718
2	115.04	2.41	2-keto-isovalerate	C00141	60	89.02	5.74	lactate	C00186|C00256|C01432
3	149.06	2.18	3-methylphenylacetic acid	HMDB02222	61	289.12	2.46	L-arginino-succinate	C03406
4	184.99	2.11	3-phosphoglycerate	C00197	62	455.25	2.84	LCA-3-SO4	LCA-3-SO4
5	119.01	1.96	3-S-methylthiopropionate	C08276	63	130.09	3.42	leucine/isoleucine	C00123
6	182.04	2.96	4-Pyridoxic acid	C00847	64	133.01	5.87	malate	C00149|C00497|C00711
7	233.09	2.43	5-methoxytryptophan	HMDB02339	65	164.04	2.65	Methionine sulfoxide	HMDB02005
8	458.19	2.09	5-methyl-THF	C00440	66	187.07	1.83	N-acetyl-glutamine	HMDB06029
9	388.95	3.70	5-phosphoribosyl-1-pyrophosphate	C00119	67	122.02	1.99	nicotinate	C00253
10	275.02	1.81	6-phospho-D-gluconate	C00345	68	146.04	2.09	O-acetyl-L-serine	C00979
11	442.14	2.94	7_8-dihydrofolate	7_8-dihydrofolate	69	319.05	2.66	Octoluse 8/1P	Octoluse 8/1P
12	257.09	5.47	acadesine	D02742	70	399.00	4.42	Octoluse Bisphosphate	Octoluse Bisphosphate
13	101.02	5.74	acetoacetate	C00164	71	131.08	3.36	ornithine	C00077
14	138.98	2.83	acetylphosphate	C00227	72	367.02	2.54	orotidine-5--phosphate	C01103
15	173.01	1.80	aconitate	C00417|C02341	73	131.00	4.47	oxaloacetate	C00036
16	145.02	4.45	a-ketoglutarate	C00026	74	218.10	8.12	pantothenate	C00864
17	157.03	3.83	allantoin	C01551	75	164.07	8.55	phenylalanine	C00079
18	160.06	8.48	Aminoadipic acid	C00956	76	165.05	8.55	Phenyllactic acid	Phenyllactic acid
19	337.06	3.46	aminoimidazole carboxamide ribonucleotide	C04677	77	145.03	1.98	Phenylpropiolic acid	HMDB00563
20	175.02	2.68	Ascorbic acid	C00072	78	163.04	2.44	phenylpyruvate	C00166
21	243.08	2.11	biotin	C00120	79	166.98	2.14	phosphoenolpyruvate	C00074
22	465.30	1.92	cholesteryl sulfate	HMDB00653	80	183.06	3.25	Phosphorylcholine	C00588
23	407.28	4.57	Cholic acid	HMDB00619	81	137.02	3.05	p-hydroxybenzoate	C00156|C00587
24	129.02	2.40	Citraconic acid	C02226	82	225.04	3.82	prephenate	C00251
25	191.02	3.90	citrate/isocitrate	C00158|C00311	83	114.05	3.64	proline	C00148
26	130.06	3.09	creatine	C00300	84	119.03	5.75	purine	C00465
27	112.05	5.36	Creatinine	C00791	85	167.08	2.59	Pyridoxamine	C00534
28	239.02	1.98	Cystine	C00491	86	168.06	3.62	pyridoxine	C00314
29	242.08	10.15	cytidine	C00475	87	128.03	9.07	Pyroglutamic acid	C01879
30	489.99	2.31	dATP	C00131	88	87.01	5.74	pyruvate	C00022
31	457.22	2.13	DCA-3-SO4	DCA-3-SO4	89	166.01	4.21	quinolinate	C03722
32	306.05	3.22	dCMP	C00239	90	375.13	1.98	riboflavin	C00255
33	250.10	2.01	deoxyadenosine	C00559	91	229.02	1.72	ribose-phosphate	C00117
34	266.09	5.60	deoxyguanosine	C00212	92	383.12	2.15	S-adenosyl-L-homocysteine	C00021
35	213.02	1.78	deoxyribose-phosphate	C00673	93	88.04	8.38	sarcosine	C00213
36	227.06	5.75	deoxyuridine	C00526	94	171.01	2.44	sn-glycerol-3-phosphate	C00093
37	199.00	2.15	D-erythrose-4-phosphate	C00279	95	117.02	9.85	succinate	C00042
38	209.03	3.81	D-glucarate	C00767	96	124.01	8.25	taurine	C00245
39	257.01	1.72	D-glucono-lactone-6-phosphate	C01236	97	514.28	6.72	TCA	TCA
40	258.04	2.01	D-glucosamine-6-phosphate	C00352	98	498.28	4.80	TCDCA	TCDCA
41	157.02	3.56	dihydroorotate	C00337	99	264.10	7.98	thiamine	C00378
42	339.00	2.62	fructose-1-6-bisphosphate	fructose-1-6-bispho	100	344.06	2.04	thiamine-phosphate	C01081
43	163.06	3.35	Fucose	Fucose	101	241.08	4.14	thymidine	C00214
44	115.00	2.38	fumarate	C00122	102	125.03	2.65	thymine	C00178
45	464.31	7.23	GCA	GCA	103	482.30	7.38	TLCA	TLCA
46	448.30	8.01	GDCA	GDCA	104	203.08	7.50	tryptophan	C00078|C00525|C00806
47	257.10	2.52	Glycerophosphocholine	C00670	105	180.06	2.04	tyrosine	C00082
48	116.05	7.53	Guanidoacetic acid	C00581	106	391.28	3.17	UDCA	UDCA
49	179.05	6.04	Hexose	Hexose	107	471.24	7.46	UDCA-3-SO4	UDCA-3-SO4
50	259.03	1.73	hexose-phosphate	C00085	108	403.00	2.08	UDP	C00015
51	131.07	4.05	Hydroxyisocaproic acid	HMDB00746	109	323.03	6.67	UMP	C00105
52	151.04	2.16	Hydroxyphenylacetic acid	C05852	110	111.02	2.75	uracil	C00106
53	179.04	2.02	hydroxyphenylpyruvate	C01179	111	167.02	4.21	Uric acid	C00366
54	130.05	8.39	hydroxyproline	C01157	112	243.06	3.22	uridine	C00299
55	135.03	4.54	hypoxanthine	C00262	113	116.07	4.26	valine	C00183
56	160.04	7.27	Indole-3-carboxylic acid	HMDB03320	114	151.02	4.50	xanthine	C00385
57	267.07	3.19	inosine	C00294	115	363.04	2.23	xanthosine-5-phosphate	C00655
58	188.04	4.54	Kynurenic acid	C01717					

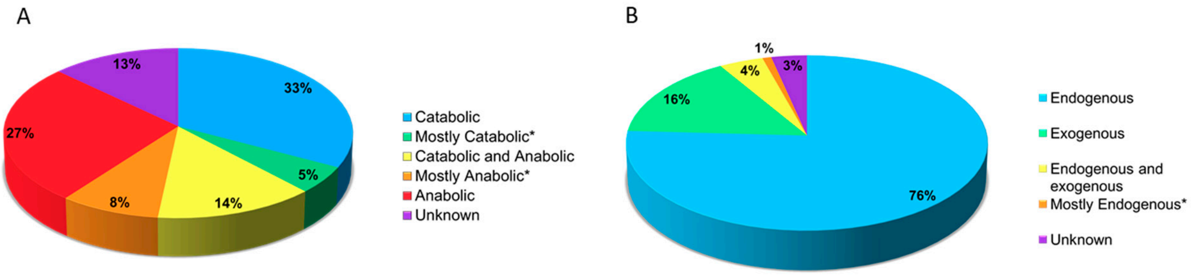

Figure S1. (**a**) Metabolic actions of 115 metabolites identified; (**b**) Ontological sources of 115 metabolites identified, taken from Human Metabolite Data Base (HMDB) (https://hmdb.ca/metabolites/).

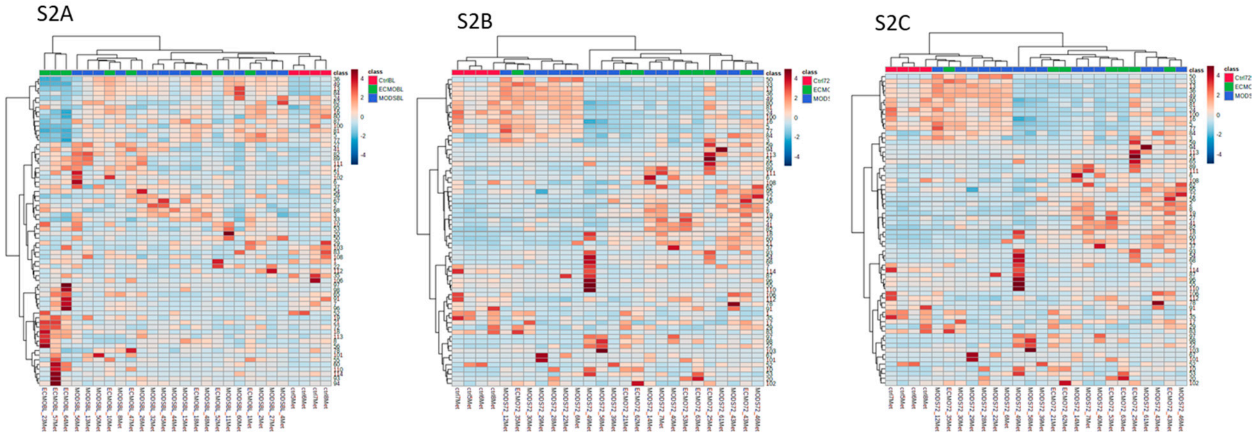

Figure S2. Clustering result shown as heatmaps at baseline (**A**), 72 hours (**B**), and 8 days (**C**).

	Pearson	Spearman	Kendall
**Time 1**			
Coefficient	0.889	0.866	0.708
Test Statistic	9.130	307.833	4.819
p-value	0.000	0.000	0.000
**Time 2**			
Coefficient	0.578	0.711	0.511
Test Statistic	3.167	511.077	3.304
p-value	0.005	0.000	0.001
**Time 3**			
Coefficient	0.825	0.850	0.684
Test Statistic	4.835	54.575	3.240
p-value	0.001	0.000	0.001

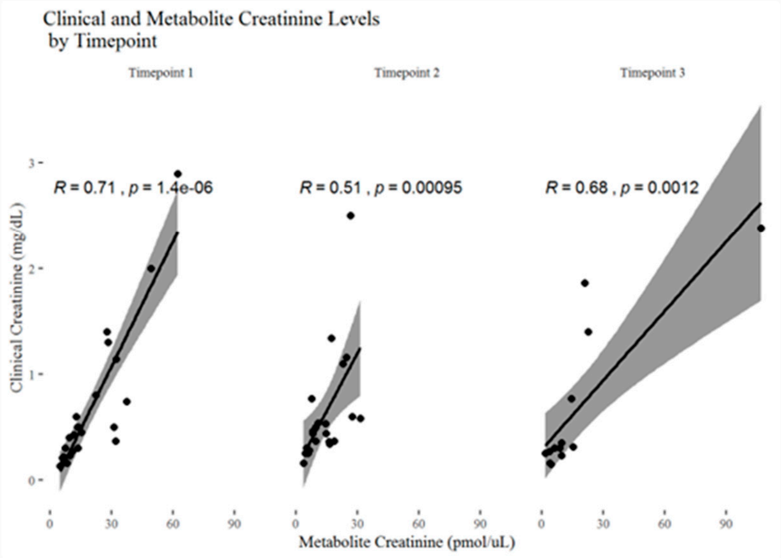

Figure S3. Correlation of clinical creatinine to untargeted metabolite value(s).
